# LPWAN-Based Vehicular Monitoring Platform with a Generic IP Network Interface

**DOI:** 10.3390/s19020264

**Published:** 2019-01-11

**Authors:** José Santa, Ramon Sanchez-Iborra, Pablo Rodriguez-Rey, Luis Bernal-Escobedo, Antonio F. Skarmeta

**Affiliations:** 1 Department of Information and Communications Engineering, University of Murcia, 30100 Murcia, Spain; ramonsanchez@um.es (R.S.-I.); rey@um.es (P.R.-R.); luis.bernal@um.es (L.B.-E.); skarmeta@um.es (A.F.S.); 2 Department of Electronics and Computer Technology, Polytechnic University of Cartagena, 30202 Cartagena, Spain

**Keywords:** ITS, vehicular communications, LoRa, LPWAN, IoT, monitoring platform

## Abstract

Remote vehicle monitoring is a field that has recently attracted the attention of both academia and industry. With the dawn of the Internet of Things (IoT) paradigm, the possibilities for performing this task have multiplied, due to the emergence of low-cost and multi-purpose monitoring devices and the evolution of wireless transmission technologies. Low Power-Wide Area Network (LPWAN) encompasses a set of IoT communication technologies that are gaining momentum, due to their highly valued features regarding transmission distance and end-device energy consumption. For that reason, in this work we present a vehicular monitoring platform enabled by LPWAN-based technology, namely Long Range Wide Area Network (LoRaWAN). Concretely, we explore the end-to-end architecture considering vehicle data retrieving by using an On-Board Diagnostics II (OBD-II) interface, their compression with a novel IETF compression scheme in order to transmit them over the constrained LoRaWAN link, and information visualization through a data server hosted in the cloud, by means of a web-based dashboard. A key advance of the proposal is the design and development of a UNIX-based network interface for LPWAN communications. The whole system has been tested in a university campus environment, showing its capabilities to remotely track vehicle status in real-time. The conducted performance evaluation also shows high levels of reliability in the transmission link, with packet delivery ratios over 95%. The platform boosts the process of monitoring vehicles, enabling a variety of services such as mechanical failure prediction and detection, fleet management, and traffic monitoring, and is extensible to light vehicles with severe power constraints.

## 1. Introduction

Monitoring systems have definitely evolved since the emergence of the Internet of Things (IoT) paradigm [1]. Old-fashion complex supervision systems are now being replaced by more recent wireless cost-effective solutions that permit control and observation of a wide range of elements or events, almost in real-time and from any place with a connection to the Internet. Many technological advances have contributed to improvements in this field, but two have been decisive for its quick implantation, namely: (i) The development of low-cost and energy-efficient monitoring devices, and (ii) the progress of communication technologies to match the requirements of these systems, such as long-range and robust transmissions.

Cheap and easy-to-use hardware platforms are now available on the market, and are reaching the general public. Example of these devices are the Arduino or Raspberry Pi boards, which are capable of performing functions that only some years ago needed industrial hardware. Thereby, these platforms allow easy monitoring of physical events by themselves, or they may be connected to larger systems with the aim of extracting monitoring data. Thereafter, the collected data are forwarded to centralized control systems, usually through wireless links. Thus, regarding the wireless transmission technologies employed in these architectures, some features are of prominent importance in order to boost the deployment of both reliable and practical monitoring systems. These characteristics are related to the need of having long-range communication solutions with very low energy consumption for end-devices, because they are usually powered by batteries. Another desirable feature is system scalability, as it is envisioned that billions of end-devices will be simultaneously connected within future IoT architectures [2]. In this line, one communication technology that meets these demands, and that is fully integrated within the IoT ecosystem since its inception, is the Low Power-Wide Area Network (LPWAN). This recently-proposed communication alternative promises long transmission distances (over 15 km in rural environments), long end-device lifetime in years, and high scalability, which permits the connection of hundreds or even thousands of end-nodes concurrently to a single base station [3]. For these reasons, LPWAN solutions are attracting the attention of both the research community and industry [4,5]. Among the different technologies following the LPWAN paradigm, LoRaWAN (Long Range Wide Area Network) has become one of the most adopted solutions, due to its flexibility and characterization possibilities [3].

One vertical that is experiencing a considerable revitalization since the appearance of IoT-related services and the upcoming 5G is the vehicular sector [6]. Under the umbrella of Intelligent Transportation Systems (ITS) and Internet of Vehicles (IoV) paradigms, many novel services will take advantage of the rich variety of connectivity options that vehicles will present in the near future [7]. The integration of different communication technologies on-board vehicles will enable the development of solutions in many areas, such as safety, green transport, goods or fleet monitoring, and traffic analysis, among others. Although some efforts were done in these directions some years ago, during the emergence of IEEE 802.11p-based technologies, the need for deploying dedicated infrastructures, together with the limited range of Vehicle-to-Vehicle (V2V) communications and the penetration rate issue, lead to a deceleration of these initiatives [8]. For that reason, the integration of a series of Radio Access Technologies (RAT) in a single on-board control system has been envisioned as a promising step in gathering all of the services and applications mentioned above, which are highly related to the concept of smart cities or, in general, smart spaces [9]. While the integration of cellular technologies in the vehicular ecosystem has been deeply studied in the past years [10,11,12], the use of LPWAN-based solutions in this area has not been exploited yet [13,14,15], and they could present a real alternative for battery-constrained transport means, such as scooters or bikes.

**Figure 1:**
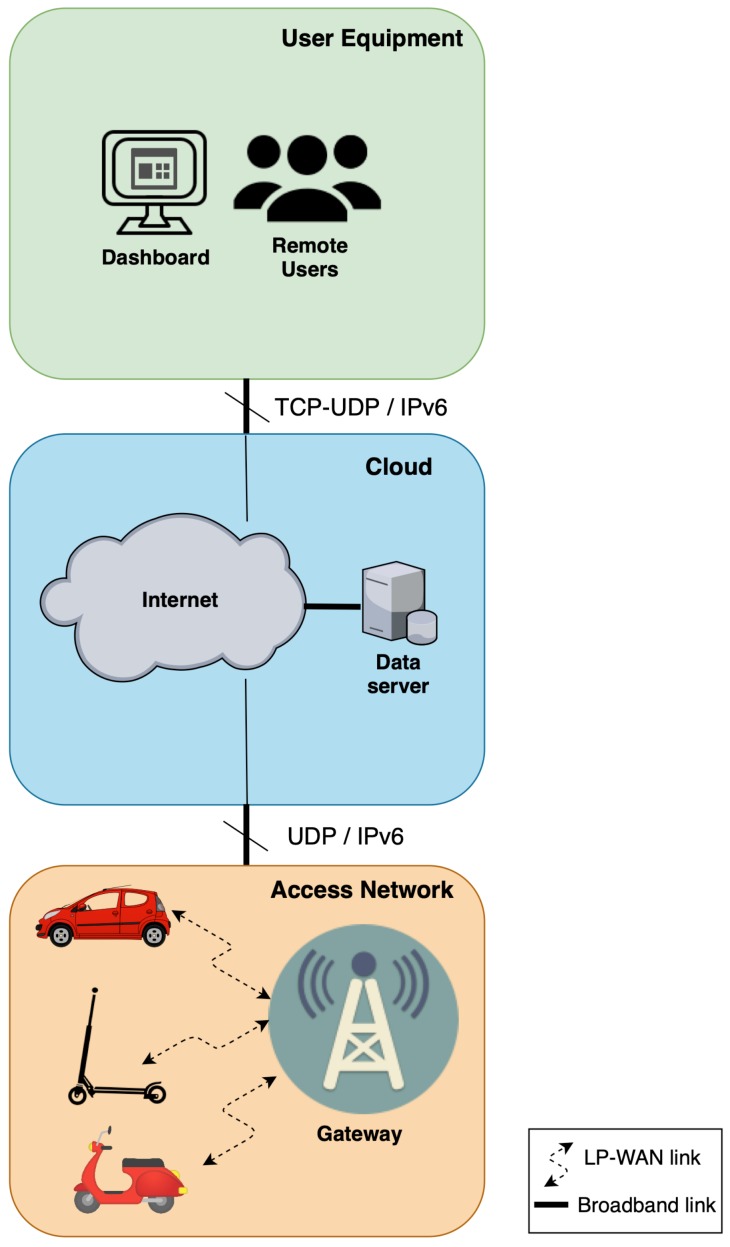
Concept of the proposal for monitoring vehicles using LPWAN.

Therefore, in this work we focus on the concept introduced in Figure 1 and propose a flexible and extensible end-to-end monitoring platform for vehicles, taking advantage of LPWAN as either the sole communication technology, or hybridized with others. We have developed the different modules of a system, that ranges from data extraction by using the On-Board Diagnostics II (OBD-II) protocol (telemetry application), to their presentation by developing a cloud-based dashboard system. As depicted in Figure 1, the data transmission from the On-Board Unit (OBU) to the infrastructure has been carried out by means of an LPWAN solution, namely LoRaWAN, with the aim of obtaining a long-distance and reliable monitoring system. In addition, we have designed and implemented a generic network interface for Linux-based systems, that permits the sending of application-layer packets through the LPWAN link using IPv6 and UDP, and using a compression mechanism based on IETF, works for these kind of networks. To the authors’ knowledge, this is the first implementation of an LPWAN-oriented network interface ready to use in normal operating systems. An experimental test-bench has been deployed, in order to demonstrate the validity of the proposed monitoring platform, which enables the tracking of vehicle position as well as mechanical status, among others. Thus, the main contributions of this work are the following: (i) A real monitoring platform for vehicles using LPWAN has been designed and developed, (ii) a performance study of LoRaWAN technology in vehicular scenarios has been conducted, and (iii) a generic Linux interface that enables direct and seamless transmission of application-layer data has been implemented.

The rest of the document is organized as follows: Section 2 reviews the related work regarding on-line monitoring systems for vehicles. An initial review of the main technologies used in the solution is given in Section 3, including LoRaWAN and IETF LPWAN compression. Next, Section 4 explores the proposed architecture. Section 5 describes the implemented development, as well as the test-bench employed for obtaining the validation results, which are showed and discussed in Section 6. Finally, the paper is concluded in Section 7, summarizing the main achievements and including future research lines.

## 2. Related Work

Monitoring vehicles, to locate or manage them, is a field that has recently been widely explored. However, most of the proposed systems are based on short- or medium-range communication technologies, such as IEEE 802.11p [15,16], cellular-based solutions [17,18], or even Radio-Frequency Identification (RFID) [19]. These transmission technologies present important drawbacks, related to their limited coverage range. In the case of short-range alternatives, such as the extensively studied IEEE 802.11p protocol, the vehicles need to support each other in order to forward the generated data toward the infrastructure elements, namely road side units (RSU), which give access to these data from the outside world (e.g., by the internet). This fact severely increases the complexity of the network management, as well as its instability due to the difficulties of 802.11p to properly work in highly dense scenarios [20]. In turn, the use of cellular-based alternatives (e.g., 4G or the upcoming 5G) has been also used for providing vehicles with seamless connectivity [21]. This technology has been especially exploited in the last ten years for fleet management purposes. However, although the previous approaches present acceptable coverage levels in urban environments [6,22], they are not able to cover large extensions per base station, especially in rural areas due to the lack of a pre-existent and expensive cellular infrastructure.

In turn, the application of LPWAN communication systems for vehicle monitoring may overcome the coverage limitations of the transmission technologies employed so far. Thus, the deployment of LPWAN networks will allow better monitoring of vehicles in remote areas, due to the long distances covered by a single gateway [4]. In fact, some European countries (such as Spain, France, and the Netherlands, among others) are now fully covered by the LPWAN solution called Sigfox [23]. Although LPWAN technologies were not designed for this specific scenario, they present interesting characteristics that make them suitable for employment in vehicular monitoring, such as great communication link budget, resilience to interference, and good performance at low power, while being robust to multi-path propagation fading and the Doppler effect. In fact, some works have evaluated LPWAN-based solutions in different network configurations valid for the vehicular ecosystem, such as single-hop [4,5], multi-hop [24,25], or Device-to-Device (D2D) [14] architectures. Thus, works in Refs. [4,5] presented two different extensive performance evaluations of LoRaWAN in different experimental scenarios, with the aim of exploring the maximum transmission distances reached depending on the environmental conditions. Authors of Ref. [4] found a maximum range of 18 km in rural areas without obstacles. In addition, authors of Ref. [5] also evidenced the robustness of LoRa against the Doppler effect. Authors of Refs. [24,25] focused on deploying multi-hop uplinks, in order to extend the communication capabilities of end-devices towards the gateway. Concretely, these works studied the optimum network routing organization, with the aim of finding the routes with the lowest energy consumption for the end-devices acting as relays. Observe that this architecture may be also valid for vehicular scenarios, as one vehicle could help another to reach the gateway. Further, considering this network topology, the number of deployed gateways may be reduced with the aim of reducing infrastructure costs. Finally, work in Ref. [14] also explored the application of LoRaWAN in vehicular scenarios, but under the opportunistic/intermittent network paradigm. The attained simulation-based results demonstrated better performance of LoRaWAN, in comparison with WiFi technology, in this type of scenario. This solution may be also valid, together with a multi-hop strategy, in order to transmit delay-tolerant data.

As a difference, with the work presented in this paper, none of the studies mentioned above deployed an end-to-end monitoring platform, neither including real data extracted from the vehicle nor a visualization system nor, as a prime advance, integrating a generic UNIX-based network interface for LPWAN. In fact, we explore the entire process as follows: (i) Vehicle data collection by means of an OBD-II interface, (ii) data compression by using the novel IETF Static Context Header Compression (SCHC) scheme, (iii) data transmission via the LoRaWAN link, through the developed LPWAN UNIX network interface, and (iv) data visualization from a data server hosted in the cloud, by means of a web-based dashboard.

## 3. Technological Background

A brief technological introduction is given first, with the aim of improving understanding of the proposal. This is focused on LoRaWAN and the compression and fragmentation mechanism for LPWAN defined by the IETF, namely SCHC.

### 3.1. LoRaWAN

LoRaWAN is one of the most prominent LPWAN-based technologies. Concretely, it is based on LoRa, which is a modulation based on the Chirp Spread Spectrum (CSS) modulation technique and developed by Semtech [26]. LoRa follows the following precepts: (i) Reduced transmission bit-rates, (ii) use of low-frequency bands (sub-GHz bands), and (iii) limited end-device communication capabilities, in order to enable long transmission distances (of more than 15 km) with reduced power consumption, that permits battery lifetimes of years [3]. Thereby, LoRa uses reduced data rates of just a few kilobits per second (kbps), which permits the development of receiving modules with high sensitivity. This fact permits the receiving equipment to decode the received signal at very low power-levels. This transmission robustness is further improved by the CSS-based modulation, as stated above [26]. In addition, LoRa uses unlicensed low-frequency bands, that is, Industrial, Medical, and Scientific (ISM) bands (such as 868 MHz and 911 MHz in Europe and America, respectively). The use of sub-GHz bands improves penetration and transmission distances, in comparison with other typical, higher-frequency, bands, such as 2.4 GHz. Aiming at saving energy in the end-devices, the communications in LoRaWAN systems are limited in both the number of transmissions per node per day, and the length of each transmission [27]. This is also related to the use of unlicensed frequency bands, so the number of transmissions and their duration need to be controlled, in order to avoid overuse by certain applications or nodes.

**Figure 2:**
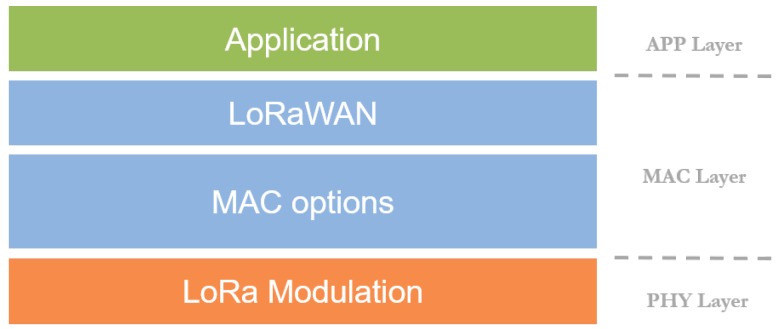
LoRaWAN stack.

**Figure 3:**
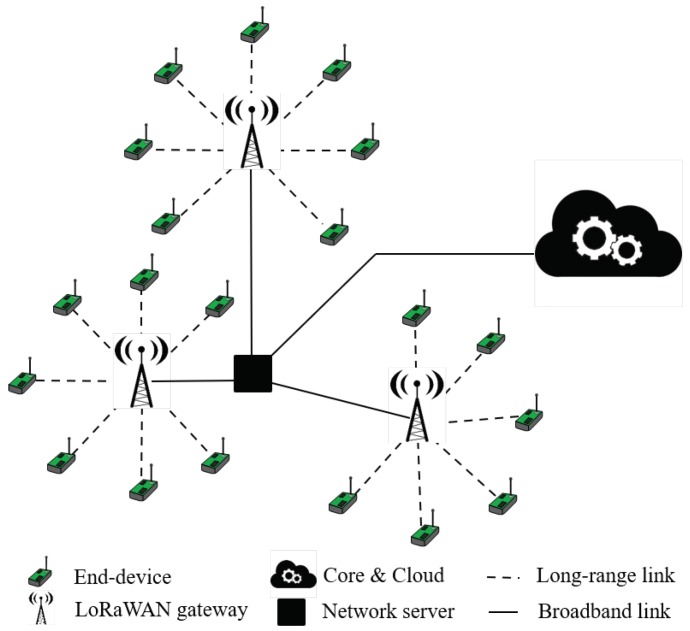
LoRaWAN network architecture.

Regarding the architecture of LoRaWAN networks, as shown in Figure 2, there is another layer on top of the PHY level (LoRa) that gives the name to the whole system, LoRaWAN. Actually, it defines the MAC-layer mechanisms and other architectural aspects, such as network security and its organization. Regarding the latter, LoRaWAN makes use of a star or star-of-stars topology (Figure 3). This architecture permits simple and direct communication between end-devices and the gateway, hence avoiding other configurations with higher complexity (such as mesh networks). Observe in Figure 3 that end-devices directly transmit to the gateway in one single hop (i.e., without the help of the rest of the network).

Considering mobility issues, end-devices transmit LoRaWAN messages in broadcast mode regardless of their position, movement, or speed. This means that any LoRaWAN gateway that hears the message will forward it to the correspondent network server (network service in Figure 3); this way, changes or hand-overs among gateways are completely seamless for end-devices.

Finally, an interesting feature of LoRaWAN is the possibility of adapting its PHY parameters, namely LoRa parameters, depending on the transmission conditions. Therefore, LoRa has three different configuration parameters: Spreading Factor (SF), Coding Rate (CR), and bandwidth (BW). The SF adjusts the spreading level of the transmitted signal, in comparison with the original one. By enlarging transmission spreading, the link robustness is increased at the expense of reducing the data-rate. The transmission robustness may be additionally increased by including redundant information in each frame. The CR defines the number of redundant bits, in comparison with the useful data, and may be tuned using different pre-defined values in the European 868 MHz ISM band. Finally, LoRa transmissions usually employ a BW of 125 kHz, but other configurations are supported as well (such as 250 kHz or 500 kHz).

### 3.2. SCHC

A key technological enabler to send IPv6 datagrams over an LPWAN network is the SCHC scheme [28], recently drafted by the IETF lpwan work-group. The base proposal enables the compression and fragmentation of IPv6/UDP packets over any LPWAN network. To this end, communication peers share a pre-provided context that indicates how to compress and decompress the packets received. This context remains static during the life-time of the device, and contains a number of compression and fragmentation rules to be applied to different kind of packets. Thus, a short RuleID that is known beforehand is included in the packet header, instead of sending longer header fields.

In the scheme considered in this work, both the vehicle on-board unit and an application gateway have to agree on the SCHC compression rule applied. Hence, this approach is mainly applicable to the LPWAN communication and packet management segment, with the great advantage of interconnecting vehicles to the Internet in the uplink and downlink directions seamlessly for both ends of the communication. This is an interesting feature, aimed at integrating LoRaWAN end-devices within the IPv6 ecosystem, increasing their communication capabilities.

The potential of SCHC was initially evaluated in our previous work in Ref. [29], providing promising results by transmitting Internet Control Message Protocol version 6 (ICMPv6) packets in a real environment. In this paper, this work is extended by addition of a data gathering platform (that uses OBD-II for the case of regular cars), a monitoring web-based interface and, above all, a remarkable development of a generic UNIX network interface to transmit IPv6/UDP packets over LPWAN.

## 4. Architecture of the Proposal

**Figure 4:**
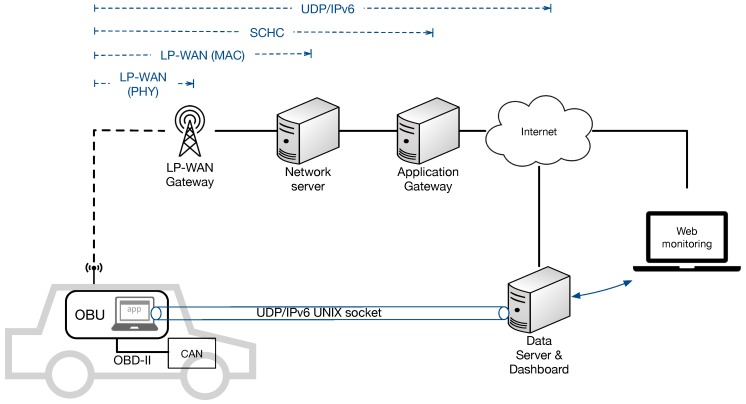
Overall system architecture.

In this Section, the details of the architecture for remote monitoring in the field of road vehicles is presented. This architecture, briefly introduced in Figure 1, and now detailed in Figure 4, comprises an LPWAN-enabled access network in which end-nodes (i.e., the vehicles) directly communicate with a communication gateway, by means of long-range links established by an LPWAN technology. This communication is abstracted to applications, thanks to a new network interface that appears to the user as a regular UNIX one. As depicted in Figure 4, this new interface acts as an LPWAN tunnel that transports UDP/IPv6 datagrams, which finally reach an application gateway after passing through the wireless LPWAN medium. The network server deals with MAC-level LPWAN packets, while the application gateway is in charge of interconnecting with IPv6 Internet and forwards the packets to a processing node in the cloud, which provides processed information to final services, or in a dashboard fashion. As can be seen in the diagram, the interconnection with IPv6 Internet is possible thanks to the use of SCHC, which adapts packets to traverse the constrained LPWAN network domain.

Although there is a direct application of the architecture in the regular car domain, it is important to emphasize the potential of the system presented to monitor new-age personal mobility vehicles, given the advantages of the synergy between embedded hardware platforms and LPWAN communications. For these vehicles, an embedded hardware platform can benefit from the solution, providing monitoring capabilities at low energy cost. Given that a great part of energy consumption in mobile devices is from the communication module, the usage of LPWAN technologies bring power savings within a new mobile IoT domain.

**Figure 5:**
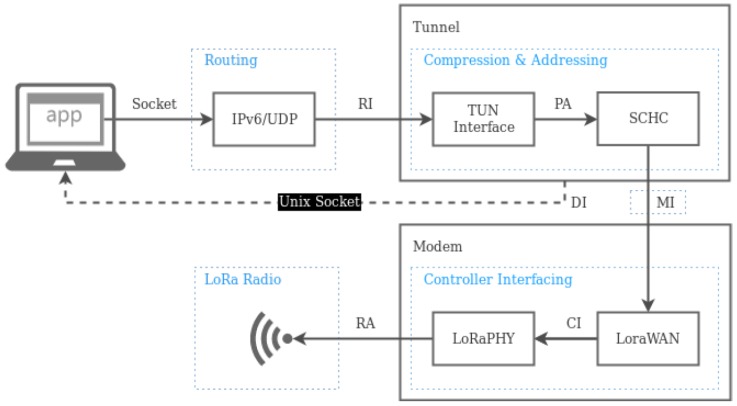
Design of the network interface solution for LPWAN.

A detailed description of the generic UNIX interface embedded in the solution is given in Figure 5. It is assumed that the on-board computer is provided with a UNIX-based operating system, and it is attached with the LPWAN modem within the same enclosure or a different one. In the diagram, LoRaWAN is showed, as it is the LPWAN technology used in the proof of concept.

The data flow starts in the OBU application, which collects data and sends them to the remote server through the internet, using the LPWAN link as a wireless last-mile network access. The data collected (or the information to be transmitted) is packed and sent using a common UNIX socket, programmed in our OBU. This is possible thanks to a new tunnel (TUN) interface, which captures the packets and adapts them to be transmitted through the LPWAN network. Our middleware compresses and fragments data using SCHC, following a context previously agreed, as explained above. Then the adapted packet, or a set of fragments, are passed to the modem for their transmission.

The design showed in Figure 5 includes a set of interfaces between the main software modules, which are detailed next:

- Socket Interface: Allows the creation of transport-level packets and their transmission through the communication stack.
- Routing Interface (RI): Applies layer-three rules to the packets received by the interface, such as filtering and routing to enter the tunnel interface.
- Packet interface (PA): Moves packets to be transmitted to the SCHC compression and framing module.
- Modem Interface (MI): Takes care of modem configuration commands and sends/receives the SCHC packets.
- Controller Interface (CI): Forwards LPWAN packets and modem configuration commands to the PHY radio unit.
- Radio interface (RA): Sends information through the radio medium using LPWAN modulation and coding.

UDP is chosen as the transport protocol for the Socket Interface, given the communication constraints imposed by LPWAN technologies, which make it impractical to consider connection-oriented protocols, such as TCP. Low data rates, an asymmetric performance of the uplink and downlink channels, and significant delays are the main reasons why datagram-based protocols are preferable. It is important to note that our network interface is designed to be adapted to different LPWAN technologies. For this, the MI interface should be implemented to communicate with the modem used, as well as the CI (if this is not implemented by the same modem). As said above, our reference implementation uses LoRaWAN, and MI uses AT commands to talk with the modem. CI is implemented with SPI communication within the LoRa transceiver.

Although the LPWAN network interface is mainly described from a sender point of view, it is important to note that the inverse process is needed when an LPWAN packet is received from the wireless medium. The RI interface prevents the OBU from receiving packets from unauthorized senders. Moreover, when an entering packet is received, a server socket is supposed to be listening in a proper port.

## 5. Test-Bench

In this section we first present the scenario and context of our validation tests. Thereafter, the equipment employed for the experimental setup is comprehensively explored.

### 5.1. Scenario

**Figure 6:**
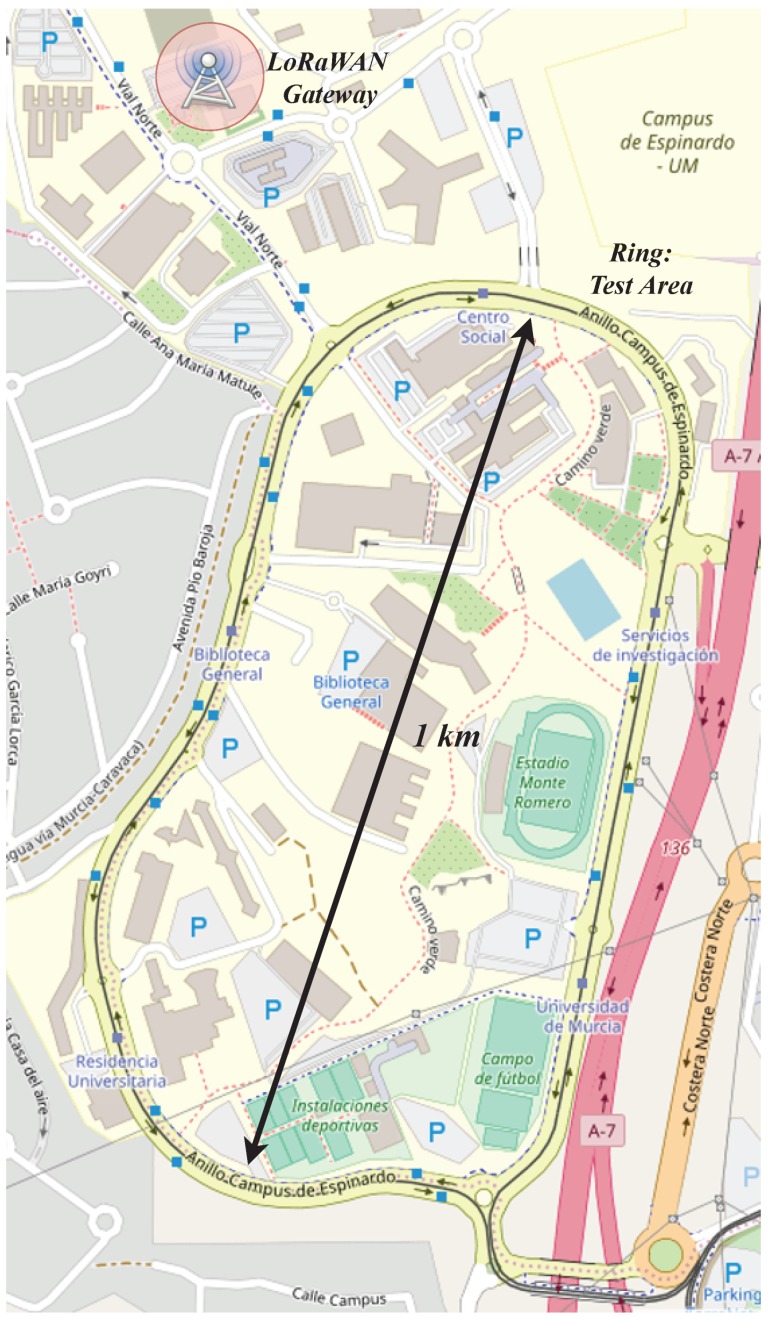
Test scenario.

As explained before, the designed and implemented system is devoted to monitor and report the current status of road vehicles. Concretely, our validation test has been contextualized in the task of monitoring the vehicles employed for providing public services within the University of Murcia (Spain) campus, e.g., postal service or internal transport. As shown in Figure 6, the main road in the campus is a ring that links the different faculties or service buildings. The major axis of this ring is 1 km long. This scenario presents high tree areas, buildings of up to five floors, and other obstacles for the wireless propagation, which convert this scenario in a realistic test-bench for our real-time vehicle monitoring platform. Please, note that the gateway location has been indicated as well in Figure 6, which was affixed on the roof of an annex building of the Computer Engineering Faculty. This is an elevated position with respect to the ring area, which was the main region of study in our tests.

Following the architecture presented in Figure 4, during the tests the data transmitted from the vehicle were received and forwarded by the gateway towards a central data server in the cloud, which processed and presented this information by means of a web-based dashboard. Moreover, the signal strength of the LoRa channel, in terms of Received Signal Strength Indicator (RSSI), was evaluated along the testing path.

Finally, regarding system scalability, please note that this work aims to present a proof of concept of the developed and deployed architecture, by using one transmitting vehicle at a time. In the case of having many vehicles concurrently transmitting data-packets to a common gateway, a planning study should be carried out, in order to estimate the number of simultaneous vehicles that can be hosted by a single gateway, depending on the frequency of the transmissions and LoRa parameters, such as SF, CR, BW, and packet length. For further information regarding LoRaWAN scalability, please refer to the detailed analyses presented in Refs. [27,30].

### 5.2. Equipment and Implementation

The system architecture presented in Figure 4 has been developed to enable the validation and testing of the proposal. This has involved both a software development and a hardware set-up. The implementation has been released as open source (https://github.com/n0p/loratun), and it is available for replicating the experiments carried out in this work, or for use in other projects.

Regarding the UNIX network interface for LPWAN, the RI interface has been implemented using the *ip* UNIX command, in order to establish the forwarding rules to enable the transmission of packets through the new interface. No packet filtering has been established for incoming communications. The PA interface has been achieved by cloning the UNIX */dev/net/tun* interface into a new tunneling interface to be controlled with *ioctl* calls. The flags *IFF_TUN* and *IFF_NO_PI* have been used in the *ioctl* interface request *ifreq*. When the packets are captured by our middleware through this new interface, they are processed and sent to the SCHC software module, which is an evolution of the implementation presented in Ref. [29] whcih now supports UDP/IPv6 compression. The MI interface with the modem is based on serial port communication, using a block-based device and an interface of type /dev/ttyUSB*, given that a USB-to-serial converter is used.

**Figure 7:**
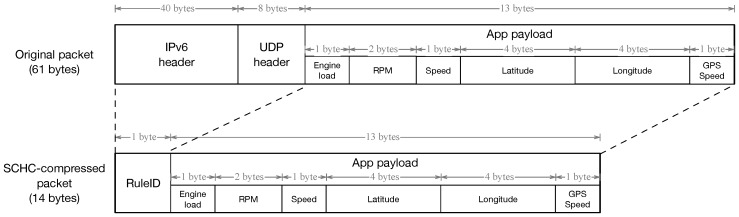
SCHC compression of data packets sent by vehicles.

As stated above, and further discussed in Ref. [31], the restrictions posed by the LPWAN link leads to the need of employing compression schemes with the aim of optimizing the size of the packets to be sent in this constrained transmission channel. To address this need, our SCHC module has been developed on the basis of the guidelines described in Ref. [28]. In SCHC, the end-node and application gateway (Figure 4) share a common pre-provided context that indicates how to manage the information contained in the protocol headers, in order to remove them from the packet sent over the LPWAN link. Thereby, Figure 7 illustrates the original packet, and the one outputted by the SCHC module of our solution. The SCHC rule applies the compression action for all of the header fields. This is possible thanks to knowing, in this case, the IPv6 addresses, UDP ports, and other static configurations of both ends (pre-provided context). This way, the 40 Bytes of the IPv6 Header, and the 8 Bytes of the UDP header, are compressed down to 1 Byte, by including a new SCHC header called RuleID, which indicates the way to proceed with the packet once it reaches the application gateway. As can be seen, the application payload remains untouched.

A regular laptop with the Ubuntu operating system was used in the tests, and was connected with the OBD-II port of a 2003 Honda Civic 1.6 i-VTEC with petrol engine. A Bluetooth GPS was used for gathering positioning data, and an ELM327-based OBD-II device was employed for obtaining car diagnosis data. The OBD-II device was connected with the laptop using Bluetooth. A software was developed, aimed at periodically obtaining the position in NMEA format and a set of OBD parameter identifiers (PIDs), and sending application-level packets with these data to the IPv6-addressed data server, by using the LPWAN UNIX interface. The communication with our UNIX interface is a regular *AF_INET6 SOCK_DGRAM* socket supporting IPv6/UDP. The data sent in the packets can be seen in Figure 7 within the application payload. From the OBD device, the engine load, RPM, and speed are used, and the latitude, longitude, and speed are taken from the GPS receiver.

In our case, we have used LoRaWAN as the communication technology, and the modem was comprised of an Arduino board provided with an RN2483 radio module by Microchip. It operates in LoRaWAN Class A mode, using the ISM 868 MHz frequency band. An ommnidirectional 2.2 dBi antenna has been attached to this device, to transmit at 14 dBm power. The other end of the LoRaWAN channel was a RisingHF RHF2S008 base-station gateway, using Semtech’s SX1301 chip. In this case, an omnidirectional antenna of 5 dBi was used.

The LoRa parameters were statically set with SF 12, CR 4/5, and BW 125 kHz; the adaptive data rate (ADR) feature of LoRaWAN was disabled for the testing activities. The rest of the configurations employed in the experimental tests are shown in Table 1. Regarding LoRaWAN security, its functions were kept untouched, and so end-to-end AES encryption was attained [26]. Please note that the encryption/decryption process was transparent for us, as it was automatically performed by the LoRaWAN modules in both the end-nodes and the gateway. Further, the joining procedure followed by the motes in order to gain connectivity through the gateway was the Over The Air Activation (OTAA), defined by the LoRaWAN specification.

Packets received by the gateway are forwarded to the network server, which deals with LoRaWAN MAC encapsulation and sends the SCHC packet to the application gateway. This is in charge of decompressing and reassembling fragments, with the aim of finally obtaining the original IPv6 datagram to be forwarded to the data server (Figure 4).

The data server receives the UDP data messages and feeds an InfluxDB database. This server also hosts a dashboard, implemented with Grafana [32], which takes data from the database to show all vehicle parameters in real-time. A set of plots, with their corresponding database queries, have been included in the interface to show, among others, the path followed by a vehicle, individual OBD parameters, or a mix of them, and alerts attending to pre-configured events or thresholds.

## 6. Results

**Figure 8:**
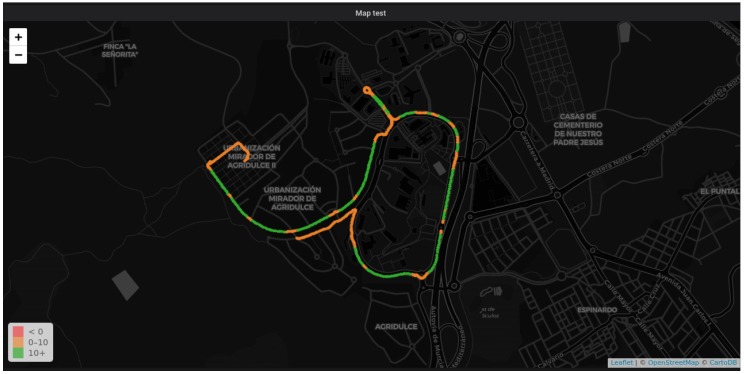
Visualization of the path followed by the vehicle in the Grafana dashboard.

**Figure 9:**
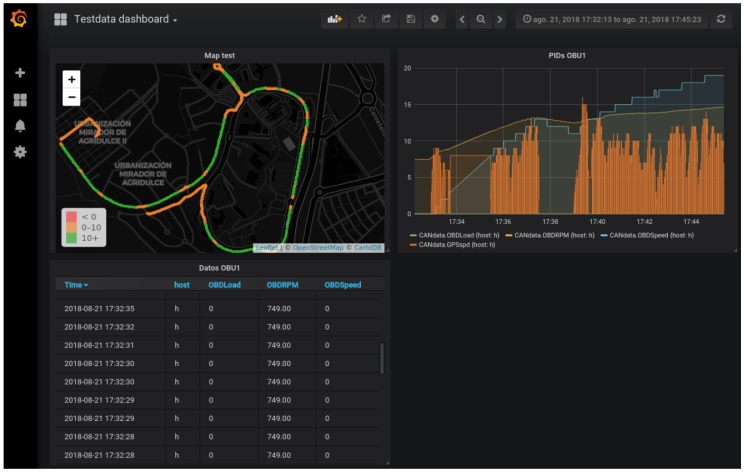
Main view of the Grafana dashboard.

A clear view of good operation of our platform can be seen in the data collected from the vehicle, both in real time and in a historical fashion. Figure 8 includes a screen-shot of the Grafana platform hosted by the data server. An OpenStreetMap component has been used to highlight the path followed by our Honda Civic within the campus. A different color is used to indicate the stretches where the car drove at higher speeds (consider that the campus has a speed limit of 20 km/h). The screen-shot in Figure 9 shows a more general view of the capabilities of the monitoring application. Here, apart from the path followed by the car, the raw parameters received and both real-time and historical records of the OBD data are shown. In the plot on the right, the engine load, revolutions per minute, and speed are shown.

**Figure 10:**
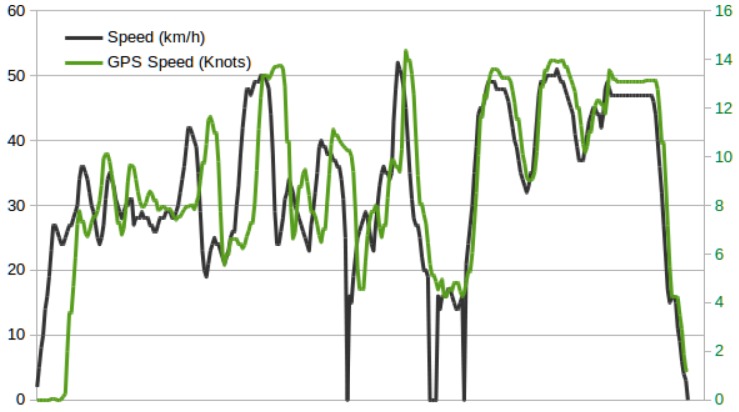
OBD and GPS speed.

An interesting issue, detected when gathering the vehicle speed, was the difference between OBD and GPS data. Figure 10 shows the speed reported in one of the tests. It can be seen that GPS speed values are delayed, as compared with the OBD data. This is attributed to two factors: The positioning algorithm, which uses cumulative data to compute fixes, and the buffering carried out by the GPS UNIX daemon used (*gpsd*), which saves the last computed position to serve requests from the OBU data gathering application.

**Figure 11:**
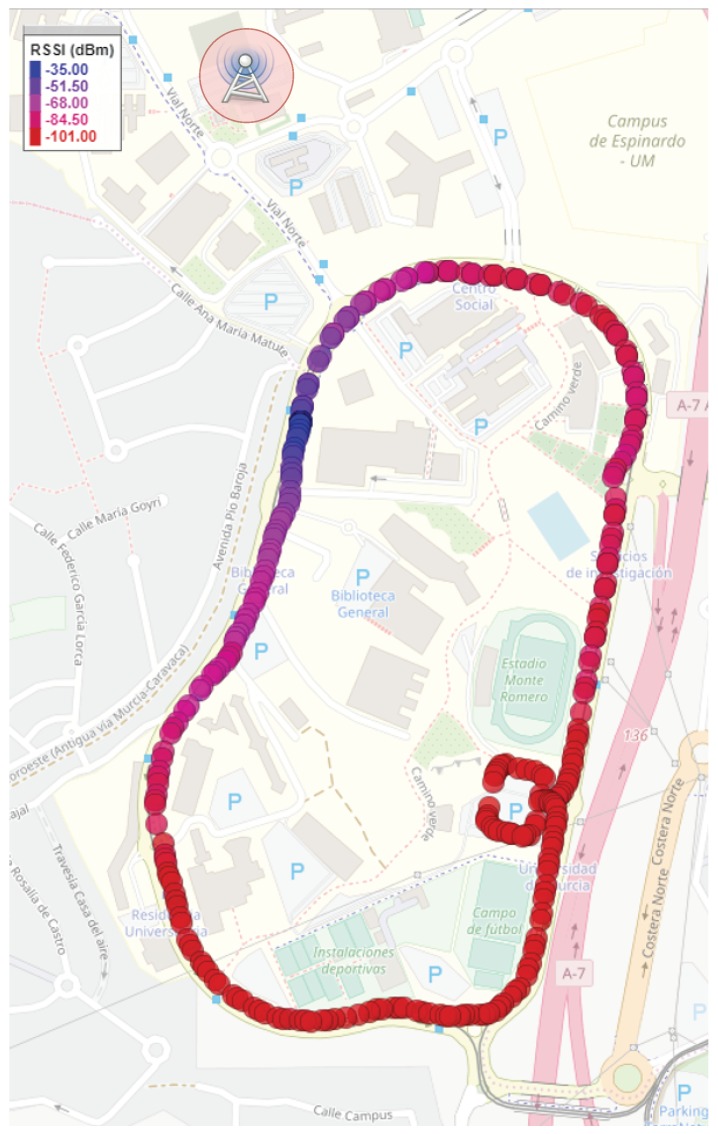
Study of the LoRaWAN signal strength.

In order to validate our proposal in a real scenario from a communications perspective, a performance evaluation of LoRaWAN was also conducted around the university campus, obtaining the results shown in Figure 11. Concretely, the LoRaWAN-enabled OBU periodically transmitted data packets to the gateway with the aim of finding non-covered zones. The RSSI, measured by the gateway in dBm, is plotted on the campus map, indicating good operation of the system around the ring. It can be seen that lower RSSI values are obtained in the lower right corner, which represents a location at 1.4 km away from the gateway. The effect of buildings is noticeable, observing the greatest RSSI values, which are not obtained at the nearest area. This is due to a group of buildings, south-east of the gateway location. In fact, as expected, the best coverage is obtained when a direct line of sight is obtained through the buildings (blue spots). Overall, observe that the lowest RSSI level attained was −101 dBm, far from the gateway receiving sensitivity; around −140 dBm.

Although it can be seen in Figure 11 that the performance level of the system is high, in terms of channel reliability (no shadow zones), the Packet Delivery Ratio (PDR) was also studied in this scenario. Thereby, with the selected LoRa configuration (SF = 12, CR = 4/5, and BW = 125 kHz), we obtained a PDR of 95%. This is an acceptable figure, which permits continuous vehicle monitoring with trustworthiness and stability. Regarding the focus of this validation study, with this deployment the institutional service vehicles can be perfectly tracked, all around the university campus. In addition, one should consider that these vehicles are usually electrically-powered; hence, they present important energetic restrictions. With the use of this LPWAN-based solution, the battery energy can be greatly saved by substituting our laptop with an embedded computer platform.

After the whole evaluation, we have checked the operation of the system and its potential to monitor vehicles using our generic LPWAN communication system. We have validated its operation, extracting OBD parameters from regular cars and GPS, showing its usefulness for such applications as fleet management or monitoring and prediction of vehicle malfunction. A low-power communication device is used as a modem and, although our on-board application is hosted by a regular laptop, our system shows an enormous potential to create embedded devices to monitor a wide range of vehicles, including power-constrained ones such as motorbikes, or even personal mobility vehicles such as bikes and scooters. This currently an on-going study, that plans to extend our work in the area [33]. Additionally, by tuning LoRa communication parameters, it is possible to reduce the data rate in favour of extending the coverage. This could be highly beneficial when large areas must be monitored using IoT communications. In any case, our deployment shows good performance at locations 1.5 km away from the gateway with the considered configuration, which means that at least an area of 7 km${}^{2}$ could be efficiently covered. In the same way, this tuning could be also applied to cover crowded areas, such as urban zones, with the aim of supporting more terminals at the expense of reducing the coverage area. Nevertheless, it is important to remark that LPWAN technologies can provide concurrent connectivity to many devices but with some limitations, given that they have emission restrictions determined by the unlicensed band duty cycles that prevent devices from flooding the network. In this line, additional work is necessary in order to estimate the number of vehicles that can be covered by a single LPWAN gateway without collision or interference-related issues. In order to face this challange, depending on the LPWAN technology employed, different solutions may be applied, such as employing different LoRa SF in the end-devices, time synchronization techniques such as in Narrow Band-Internet of Things (NB-IoT), and so on.

## 7. Conclusions

Intelligent transportation systems are experiencing a big revolution, due to the emergence of novel communication paradigms. One of these technologies gaining momentum is LPWAN, which can boost the synergy between ITS and IoT. Following this line, in this work we have presented an end-to-end monitoring platform for vehicles, based on LPWAN and following a data collection architecture, that enables the provision of a number of ITS services.

The platform presented involves a holistic solution that covers the next stages of a new-age ITS monitoring solution:

- Vehicle data extraction, by means of an OBD-II interface.
- Data compression, using a state-of-the-art IETF compression scheme, SCHC.
- Data delivery through an LPWAN link, using an LPWAN-capable UNIX network interface implemented from scratch.
- Storage of the retrieved data in a cloud data server, and presentation of information to end-users by means of a web-based dashboard.

The attained results confirm the validity of the proposal, which permits successful remote monitoring, almost in real-time, of vehicle position and other metrics such as its mechanical status. A validation test has been conducted in a real scenario, showing the full coverage of a university campus with transmission distances up to 1.5 km. Besides the real LoRaWAN architecture deployed to perform the experiments, it is also worth noting the implementation of the generic network interface for UNIX systems, which allows the seamless transmission of application-layer UDP/IPv6 packets through the LPWAN link. To the authors’ knowledge, this is the first development of a network interface that permits the direct transmission of data over an LPWAN-based technology, for a general-purpose operative system. In addition, the reliability of the communications over this constrained link has been studied as well, obtaining notable packet delivery ratios over 95% under motion conditions.

Future research lines include the study of this proposal in other environments, such as highways or dense urban areas, with a multitude of vehicles involved in the experiments. The UNIX LPWAN interface will be exploited in our active research lines in the fields of packet compression, fragmentation, and security for LPWAN.

## Figures and Tables

**Table 1:** Transmission parameters.

Parameter	Value
Transmission power	14 dBm
Spreading Factor (SF)	12
Coding Rate (CR)	4/5
Bandwidth (BW)	125 kHz
Bit-rate	239 bps
Packet length	14 Bytes
Packet rate	0.33 packets per second
Adaptive Data Rate (ADR)	No
Retransmissions	No

## Abbreviations

The following abbreviations are used in this manuscript:

BW	Bandwidth
CI	Controller Interface
CR	Coding Rate
CSS	Chirp Spread Spectrum
D2D	Device-to-Device
DTN	Delay Tolerant Network
GPS	Global Positioning System
GSM	Global System for Mobile communications
ITS	Intelligent Transportation Systems
ICMPv6	Internet Control Message Protocol version 6
IoV	Internet of Vehicles
IoT	Internet of Things
IPv6	Internet Protocol version 6
ISM	Industrial, Scientific, and Medical
LPWAN	Low Power-Wide Area Network
LoRaWAN	Long Range Wide Area Network
MI	Modem Interface
OBD-II	On-Board Diagnostics II
OBU	Onboard Unit
PA	Packet interface
PDR	Packet Delivery Ratio
RA	Radio interface
RAT	Radio Access Technology
RFID	Radio-Frequency Identification
RI	Routing Interface
RSU	Road Side Unit
RSSI	Received Signal Strength Indicator
SCHC	Static Context Header Compression
SF	Spreading Factor
UDP	User Datagram Protocol
V2V	Vehicle to Vehicle
